# Characterization of key off-flavor compounds in soybean and their dynamic changes during progressive processing

**DOI:** 10.3389/fnut.2026.1866732

**Published:** 2026-06-24

**Authors:** Jiaying Zhou, Danni Huang, Shijie Xu, Jian Li, Jun Liu, Yuhui Li, Wentao Zhang

**Affiliations:** 1 Key Laboratory of Green and Low-Carbon Processing Technology for Plant-based Food of China National Light Industry Council, School of Food and Health, Beijing Technology and Business University, Beijing, China; 2 China Tobacco Shandong Industrial Co., Ltd., Jinan, China; 3 Shandong Yuwang Ecological Food Industry Co., Ltd., Dezhou, Shandong, China; 4 Tobacco Research Institute, Chinese Academy of Agricultural Sciences, Qingdao, Shandong, China

**Keywords:** aroma dilution analysis, key off-flavor compounds, normal and high oleic acid soybeans, soybean-based food, stable isotope dilution analysis

## Abstract

This study systematically identifies key off-flavor compounds in normal soybean flour (NOR-SF), thereby profiling their dynamic changes among SF-based products manufactured from NOR and high oleic acid (HOA) soybeans. A total of 55 volatile compounds were detected in NOR-SF using headspace solid-phase microextraction gas chromatography mass spectrometry (HS-SPME-GC-MS), of which 18 were identified as odor-active compounds by GC-O. A total of 13 odorants with odor intensity (AI) ≥ 2 were re-quantified by stable isotope dilution analysis (SIDA), which led to the identification of 10 key odorants with flavor dilution (FD) factors ranging from 4 to 64 and odor activity values (OAVs) > 1. Odor recombination and omission experiments further confirmed their significant contributions to the off-flavor profile of NOR-SF (*p* < 0.05). Subsequently, a precise SIDA quantification of key off-flavor odorants in eight soybean-based products from two soybean varieties, combined with orthogonal partial least-squares discriminant analysis (OPLS-DA), showed that, except for heptanal, nonanal, and octanal, the concentrations of off-flavor compounds in HOA samples were lower than those in NOR samples. Moreover, the concentrations of off-flavor compounds in soybean meal and soy protein isolate were generally lower than those in soybean flour, except for 2-pentylfuran and 1-hexanol, whereas the concentrations of off-flavor compounds in textured soy protein obtained after extrusion exhibited dynamic fluctuations. These results indicate that the soybean variety significantly affects the formation and evolution of off-flavor compounds in different soybean-based products. This study provides a theoretical basis for controlling off-flavors in products derived from NOR and HOA.

## 1 Introduction

Soybean is one of the world’s most important legume crops and is nutrient-dense, containing approximately 40% protein. It provides high-quality plant proteins that are comparable to those from animal sources (1). Soybean protein is widely used to produce soybean-based food because of its stable supply, favorable functionality, and relatively low cost. However, the undesirable flavors generated during soybean processing remain a major barrier to consumer acceptance of these products (2), thereby limiting improvements in product quality and market acceptance. Lipid oxidation is one of the main pathways responsible for these off-flavors (3). Normal soybeans (NOR) contain relatively high levels of polyunsaturated fatty acids, such as linoleic acid and linolenic acid (4). These fatty acids are prone to oxidative cleavage during processing, leading to the formation of typical off-flavor compounds, including hexanal, 2-pentylfuran, and 1-octen-3-ol (5, 6). Based on this pathway, differences in fatty acid composition among soybean varieties can further influence the formation of off-flavors. Oleic acid, a monounsaturated fatty acid, typically constitutes 20–25% of the total fatty acids in NOR. Through genetic engineering, high oleic acid (HOA) varieties have been developed, in which oleic acid accounts for 75–85% of the total fatty acids (7). Compared to normal soybeans, HOA soybeans have a modified fatty acid composition, with higher levels of oleic acid and lower levels of linoleic and linolenic acids (8, 9). This composition of HOA soybean material can improve oxidative stability (10, 11), influence processing properties (12), and reduce the formation of beany off-flavors during soy-based food processing (13, 14). Therefore, comparing NOR and HOA soybeans in terms of flavor formation, particularly regarding the generation of off-flavor compounds, is important for clarifying how fatty acid composition regulates off-flavor development in soybean products. This comparison can also provide a basis for developing soybean-based foods with improved flavor quality.

With the development of deep soybean processing, soybeans have been widely processed into a variety of products, including soybean meal (SM), soy protein isolate (SPI), and textured soy protein, and are extensively used in the food industry. They differ substantially in composition, processing history, and matrix characteristics (15–17). Flavor formation during processing is a stage-dependent and cumulative process. Each processing stage can induce changes in lipid status, protein conformation, and volatile compound composition (6). Different processing operations not only alter the original physicochemical state of soybean constituents but also reshape the formation pathways and accumulation patterns of off-flavor compounds (5). However, research on the evolution of off-flavors during soybean-based food processing remains limited, and the majority of studies have focused on SPIs. Less attention has been paid to the dynamic changes in off-flavor compounds throughout the continuous processing chain, from soybean flour and soybean meal to SPI and textured soy protein. In a recent study, Xu et al. (18) employed a molecular sensory science approach combined with sensory evaluation to identify eight characteristic odorants in SPIs, including hexanal, heptanal, octanal, nonanal, (E)-2-nonenal, (E,Z)-2,6-nonadienal, (E,E)-2,4-nonadienal, and (E,E)-2,4-decadienal, which contribute to grassy and related flavor notes. They further profiled each compound and integrated the results into a comprehensive flavor map (18). Meanwhile, existing studies on off-flavor control have mainly targeted individual processing stages. For example, many studies have focused on SPI and used physical or chemical treatments to modify protein structure, thereby reducing the retention or release of off-flavor compounds. Kong et al. (6) reported that synergistic ultrasonic-thermal treatment significantly reduced the concentrations of major volatiles in SPIs, likely by altering the distribution of secondary and tertiary protein structures and strengthening protein–flavor binding. Li et al. (19) found that pretreatment with basic amino acids before spray drying promotes the exposure of hydrophobic groups to the aqueous phase, facilitates adsorption of SPIs at the oil–water interface, and increases the affinity between beany odorants and protein, which substantially reduces their release; an additive concentration of 0.3% achieved the greatest suppression of beany notes. Although these approaches provide useful strategies for flavor improvement, they do not fully explain how off-flavors are formed, transformed, and accumulated throughout the entire soybean processing chain. This limitation makes it challenging to identify the key stages responsible for off-flavor formation. As a result, subsequent flavor control still largely depends on end-point masking or flavor compensation, rather than precise regulation at raw-material and processing levels. Therefore, systematically comparing key off-flavor compounds across different soybean-based products would help elucidate the dynamic evolution of off-flavor formation during soybean processing. Tracking the formation and changes of these key off-flavor compounds can further help accurately identify their sources and determine the critical processing stages responsible for their accumulation.

This study aimed to systematically elucidate the formation and evolution of key off-flavor compounds during NOR and HOA soybean processing. The major contributors to the off-flavor profile of normal soybean flour (NOR-SF) were screened using a combination of GC–O, revised aroma dilution analysis (ADA), and odor activity values (OAVs). The key beany off-flavor compounds were further accurately quantified by stable isotope dilution analysis (SIDA). On this basis, the changes in key off-flavor compounds across different processing stages, including soybean flour, SM, SPI, and textured soy protein, as well as the differences between NOR- and HOA-soybeans, were further compared, with the aim of providing theoretical support for elucidating the mechanisms of off-flavor formation in soybean products and improving their flavor quality.

## 2 Materials and methods

### 2.1 Soybean samples

NOR and HOA soybeans were cultivated and harvested from a local farm (37.45° N, 116.37° E, Dezhou City, Shandong Province, China) in August 2021. The raw soybeans were step-by-step processed into soy flour (SF), SM, SPI, and texturized soy protein (TSP). All samples were provided by Yuwang Ecological Food Industry Co., Ltd. (Shandong, China).

### 2.2 Chemicals

n-Alkanes (C7 − C30, 98%) and 2-methyl-3-heptanone (99%) were purchased from Sigma-Aldrich Chemical Co., Milwaukee, WI, USA. Benzaldehyde (99.5%), 2,3-butanediol (98%), and methanol (>99%) were purchased from Shanghai Macklin Biochemical Co., Ltd., Shanghai, China. Hexanal (>98%), octanal (>98%), nonanal (>95%), heptanal (>95%), (E)-2-octenal (>96%), (E)-2-hexenal (>97%), 3-methyl-1-butanol (>99%), 2-pentylfuran (>98%), 1-hexanol (>98%), 1-nonanol (>99%), 1-pentanol (>99%), 1-heptanol (>98%), 2-heptanone (>98%), 1-octen-3-ol (>98%), 2-octanone (>98%), 2-ethylfuran (>98%), (E)-2-nonenal (>95%), and (E, E)-2,4-decadienal (>90%) were acquired from Tokyo Chemical Industry, Japan. d12-Hexanal (96%), d5-2-heptanone (98%), d14-heptanal (98%), d11-2-pentylfuran (98%), d11-1-pentanol (98%), d16-octanal (95%), d18-nonanal (95%), d15-1-hexanol (98%), d2-(E)-2-octenal (98%), d3-1-octen-3-ol (95%), d6-benzaldehyde (98%), d2-(E)-2-nonenal (98%), and d4-1-nonanol (98%) were provided by ZZBIO Co., Ltd., Shanghai, China.

### 2.3 Extraction of volatile compounds from the SF samples by HS-SPME

The SF samples, manufactured from NOR and HOA, were ground (FW100 high-speed grinder, Tianjin Taisite Instrument Co., Ltd., China) and sieved through a 100-mesh screen. Headspace solid-phase microextraction (HS-SPME) was employed to isolate the volatile compounds of soybean samples, following the method of Bi et al. (20) with slight modifications. For each sample, 0.8 g of SF, SM, SPI, and TSP, were added to a 20-mL headspace bottle (ANPEL Laboratory Technologies, Inc., Shanghai, China) containing 1.0 g of sodium chloride and 5 mL of deionized water, and supplemented with 2.5 μL of 2-methyl-3-heptanone (81.6 μg/mL in methanol) as an internal standard. The sample was mixed thoroughly and incubated at 50 °C for 15 min to reach equilibrium. Then, a 2-cm divinylbenzene/Carboxen™/polydimethylsiloxane (DVB/CAR/PDMS) SPME fiber was introduced and exposed to the headspace environment at 50 °C for 15 min. After extraction, the fiber was inserted into the injection port of a gas chromatography–mass spectrometric (GC–MS) system and desorbed at 250 °C for 5 min.

### 2.4 Gas chromatography–mass spectrometry analysis

A 7890B-5977A GC–MS system (Agilent Technologies, Santa Clara, CA, USA) was used to identify the volatile compounds in SF. Headspace extracts obtained by HS-SPME were analyzed on a polar DB-Wax fused-silica capillary column (60 m × 0.25 mm i.d., 0.25 μm film thickness; Agilent Technologies). The depth of the SPME fiber insertion in the injector was 14 mm. The oven program was set at 40 °C for 0.5 min. It was then increased to 140 °C at 2 °C/min, followed by a further increase to 230 °C at 10 °C/min, which was maintained for 5 min (21). Helium (purity ≥ 99.9999%; Beijing AP BAIF Gases Industry Co., Ltd., Beijing, China) was used as the carrier gas at a constant flow rate of 3.0751 mL/min. The MS was operated in full scan mode (45–450 *m*/*z*) with electron-impact ionization at 70 eV, and the ion source and interface temperatures were 200 °C and 250 °C, respectively.

### 2.5 Gas chromatography-olfactory analysis

Volatile compounds were identified using a combination strategy based on MS patterns, linear retention indices (LRIs), olfactometry, and retention time. Compounds with a match score ≥ 800 were tentatively identified by comparing their MS data with those available in the NIST 17 library (22). LRIs were calculated using n-alkanes (C7 − C30, diluted 10,000-fold with methanol) as standards under identical GC–MS conditions (23). A compound was considered a candidate if the difference between the calculated and published LRI was < 20 (24). The candidate was further confirmed by comparing odor description and retention time with its reference standard (diluted in methanol at 100-fold above the odor threshold in water).

### 2.6 Quantitation of volatile compounds of the HS-SPME extracts

A collaborative approach was applied to quantify the volatiles of soybean samples. According to an earlier study (25), the compounds identified in HS-SPME extracts were preliminarily quantified by an internal standard method (2-methyl-3-heptanone). Among them, 13 major odor-active off-flavor compounds (AI ≥ 2) were requantified through the stable isotope dilution assay (SIDA). To prepare standard solutions, isotope-labeled markers were mixed with the corresponding unlabeled standards in methanol at seven concentration ratios (1:8 to 8:1). For each ratio, 100 μL of the mixture was transferred to a 2 mL screw-cap vials. The sample was mixed with 4 μL of the isotope-labeled and unlabeled analytes followed by GC–MS analysis in the selected ion monitoring (SIM) mode. The fragment formation was detailed in Table 1 as the supporting information. Furthermore, the calibration curve was fitted with the ratio of the peak areas of isolate-labeled and unlabeled compounds as the ordinate and the ratio of concentrations as the abscissa. The limit of detection (LOD) and limit of quantification (LOQ) were determined as 3.3 and 10 b/a, respectively (26). The stable isotope-labeled internal standards were added before sample equilibration and extraction, allowing them to undergo the same pretreatment, HS-SPME extraction, desorption, and GC–MS analysis procedures as the corresponding native analytes (27, 28). This approach was used to compensate for analyte loss and matrix effects during quantitative analysis (29–34). The concentration of each compound was calculated based on the following equation (35):


$$\rho_{x}=\mathrm{Rf}\frac{A_{x}}{A_{s}}\rho_{s}$$

where *Rf* is the MS response factor of the compound relative to isotope-labeled internal standards calculated by the slope of the calibration curve; *ρ_x_* and *A_x_* are the concentration and chromatographic peak area of the target analyte, respectively; and *ρ_s_* and *A_s_* are the concentration and chromatographic peak area of each isotope markers, respectively.

**Table 1:** Identification of the major odor-active off-flavors in NOR-SF by FD and OAV.

No.	Odor-active off-flavor compounds	Standard curve	Calibration range (ng/g)^a^	*R*	*R*_f_ ^b^	LOD (ng/g)	LOQ (ng/g)	Standard selected ions^c^	Isotope selected ions^c^	Concentrations (ng/g)	FD	Odor threshold (ng/g)^d^	OAV
1	Hexanal	*y* = 1.1955*x* − 0.0328	562.42–38245.64	0.9993	1.1955	0.09	0.27	56, 72, 82	64, 80, 92	37485.16 ± 3679.37	64	5.00	7,497
2	2-Heptanone	*y* = 1.3943*x* − 0.1777	4.10–256.90	0.9973	1.3943	0.07	0.20	58, 99, 114	48, 80, 108	84.92 ± 12.82	–	140.00	0.61
3	Heptanal	*y* = 1.7674*x* − 0.0964	4.21–255.91	0.9990	1.7674	0.41	1.24	41, 55, 70	63, 75, 119	88.54 ± 5.31	4	2.80	32
4	2-Pentylfuran	*y* = 0.342*x* + 0.0901	9.78–3089.99	0.9915	0.3420	0.87	2.63	81, 82, 138	83, 85, 149	429.5 ± 17.18	8	5.80	74
5	1-Pentanol	*y* = 2.6206*x* − 0.1675	54.38–3402.31	0.9988	2.6206	0.21	0.64	42, 55, 70	48, 62, 80	3141.79 ± 125.9	4	150.20	21
6	Octanal	*y* = 2.8364*x* − 0.2318	10.24–640.53	0.9991	2.8364	0.41	1.24	69, 84, 110	78, 96, 112	212.04 ± 9.32	8	0.80	265
7	1-Hexanol	*y* = 1.2976*x* − 0.1184	55.78–3466.98	0.9990	1.2976	0.30	0.91	56, 69, 84	61, 73, 89	1699.67 ± 99.2	16	5.60	304
8	Nonanal	*y* = 0.5303*x* + 0.1892	40.01–2522.79	0.9922	0.5303	1.18	3.57	82, 98, 114	80, 112, 128	348.05 ± 13.25	32	1.10	316
9	(E)-2-Octenal	*y* = 2.1234*x* + 0.7854	20.32–1988.45	0.9969	2.1234	1.22	3.70	82, 97, 108	59, 72, 85	1622.18 ± 71.74	16	3.00	541
10	1-Octen-3-ol	*y* = 0.1897*x* + 0.1534	10.10–7662.30	0.9923	0.1897	2.67	8.09	57, 72, 85	60, 75, 88	1783.51 ± 293.43	8	1.50	1,189
11	Benzaldehyde	*y* = 1.1623*x* + 0.0814	87.40–523.95	0.9997	1.1623	0.23	0.70	77, 105, 106	82, 110, 112	224.27 ± 24.95	–	750.89	0.30
12	(E)-2-Nonenal	*y* = 18.661*x* + 7.0768	236.21–13253.25	0.9941	18.661	1.25	3.79	70, 96, 111	72, 85, 127	4173.05 ± 662.92	4	0.19	21,963
13	1-Nonanol	*y* = 0.2526*x* − 0.0288	2.61–129.03	0.9977	0.2526	0.38	1.14	70, 83, 97	58, 73, 102	32.76 ± 3.03	–	45.50	0.72

a Range of each compound concentration for the standard curve.

b Response factor (*Rf*) was the slope of the calibration curve for each isotope marker.

c Ions selected for quantitative analysis with SIM mode.

d Odor threshold obtained from van Gemert.

### 2.7 Gas chromatography-olfactory-mass spectrometry analysis

A 9100 Sniffer olfactory detection port (ODP, Brechbühler, Schlieren, Switzerland) coupled to the GC–MS system was utilized to discriminate the odor-active off-flavors in HS-SPME isolates. At the end of the capillary column, the effluent was split equally by volume between the MS detector and the ODP. The ODP transfer line was maintained at 200 °C. Humidified air stream was supplied to the sniffing port at 45 mL/min to convey the odorants. The other working conditions of GC–MS system were programmed as described in section 2.4.

Building upon the sensory panel as mentioned in section 2.10, four judges with >300 h of olfactory analysis were recruited to conduct the olfactory analysis in duplicate. The assessor was asked to detail the odor events, including sniffing time, odor description, odor intensity (AI), and odor pleasantness. The assessor was asked to rate the AI of candidates from 0 to 4, where 0 denoted no intensity, 1 denoted low intensity, 2 denoted moderate intensity, 3 denoted high intensity, and 4 denoted very high intensity (20). Volatile compound with AI ≥ 2 was considered as odor-active off-flavors. Furthermore, a revised ADA was performed to evaluate the aroma activity of volatiles in HS-SPME extracts, as described by Nedele et al. with minor modifications (36). In contrast to conventional AEDA, which is generally based on serial dilution of aroma extracts, revised ADA achieves odor dilution by changing the GC injector split ratio in HS-SPME-GC-O analysis. This approach has been used in previous studies and is considered appropriate for determining FD factors when volatile compounds are introduced by HS-SPME (37–39). Column effluent was divided by volume between the MS detector and the ODP at double increase of inlet split ratios. In particular, the inlet split ratio was set as split-less mode, followed by 2:1, 4:1, 8:1, …, 2n:1. Flavor dilution (FD) factor was therefore defined as the highest inlet split ratio, when the odorant cannot be recognized by the judges in olfactory analysis. If an odorant has a FD ≥ 2, it is identified as a major odor-active off-flavor (40).

### 2.8 Calculation of odor activity values

The OAV of volatile compound is equal to its concentration divided by its threshold concentration in water (41). Volatile compound with OAVs ≥ 1 is perceived as the potential contributor to the odor profile. In view of the matrix effect on odor perception, only the OAVs of 13 major odor-active off-flavor compounds were calculated basing on the SIDA quantification, and used to re-check the odor activity.

### 2.9 Odor recombination and odor omission studies

To confirm the identification of odor-active off-flavors by OAVs and GC-O-MS analysis, an odor recombination model was prepared for comparison with the original odor profile of SF samples. Considering the matrix effect on odor release, whey protein was chosen as the simulated protein matrix for odor recombination. This was because the deodorization of soybean flour may alter the structural properties and thus affect interactions between volatile compounds and the matrix. As a low-background protein matrix, the whey protein at the same concentration (0.8 g/mL) has been tested by GC-O-MS to ensure no flavor impact. Although this model cannot fully reproduce volatile release in the native soybean flour system, it remains suitable for verifying key off-flavor compounds, and similar approaches have been reported in related studies (42). Among the 13 odor-active off-flavors of SF samples (Table 1), 10 major odor-active off-flavors (OAVs ≥ 1 and FD ≥ 2) were mixed with the blank matrix according to their quantitative results of SIDA, followed by equilibration at 35 °C for 1 h.

To further screen the key odor-active off-flavors, 10 odor omission models were prepared in the same way as odor recombination model but omitting specified components one by one. Triangle test was conducted to statistically evaluate the difference between the omission sample and the reconstituted sample (14). For each test, the assessor was asked to sniff and recognize the difference among the samples, comprising of one odor omission model and two odor recombination models.

### 2.10 Sensory evaluation

A sensory panel consisting of 12 nonsmoking judges (six men and six women, aged 23–38 years) was selected and trained under the guidelines of ISO 8586, with the technical assistance of Agriculture and Food Standardization Institute, China National Institute of Standardization. Quantitative descriptive analysis (QDA) was performed to characterize the odor profile of SF samples and odor recombination model. Six odor descriptors, namely, grassy (hexanal, 250 μg/L), fatty ((E, E)-2,4-decadienal, 1 μg/L), fruity (1-hexanol, 280 μg/L), earthy (2-ethylfuran, 500 μg/L), musty (1-octen-3-ol, 75 μg/L), and creamy (2,3-butanediol, 1,000 μg/L), were proposed based on pre-evaluation and earlier studies by the authors (34, 41, 43). The odor standard references were dissolved in simulated protein matrix with cosolvent of 1,2-propanediol, at a concentration of 50 times above the respective odor threshold in water. The assessors were asked to rate the descriptor using an 8-point scale from 0 (not perceivable) to 9 (strongly perceivable). The results of three independent measurements for each descriptor were averaged and plotted in a spider web chart.

### 2.11 Dynamic changes in key odor-active off-flavors throughout the process of SF

To track the alteration of off-flavors during soybean process, SIDA was introduced to assay the 10 key odor-active off-flavors among SF, SM, SPI, and TSP samples. All these samples were pretreated and analyzed as described above.

### 2.12 Statistical analysis

The least significant difference among three independent sample groups was analyzed using one-way analysis of variance at the 5% significance level (RStudio 2023, RStudio Team, Boston, MA, USA). Orthogonal partial least-squares discriminant analysis (OPLS-DA) was performed by the SIMCA 14.1 (Sartorius Group, Göttingen, Germany). Data were visualized through Microsoft Excel 2019 (Microsoft Corporation, Redmond, WA, USA) and Origin 2021 (OriginLab Corporation, Northampton, MA, USA).

## 3 Results and discussion

### 3.1 Odor profile of NOR-SF and HOA-SF

**Figure 1:**
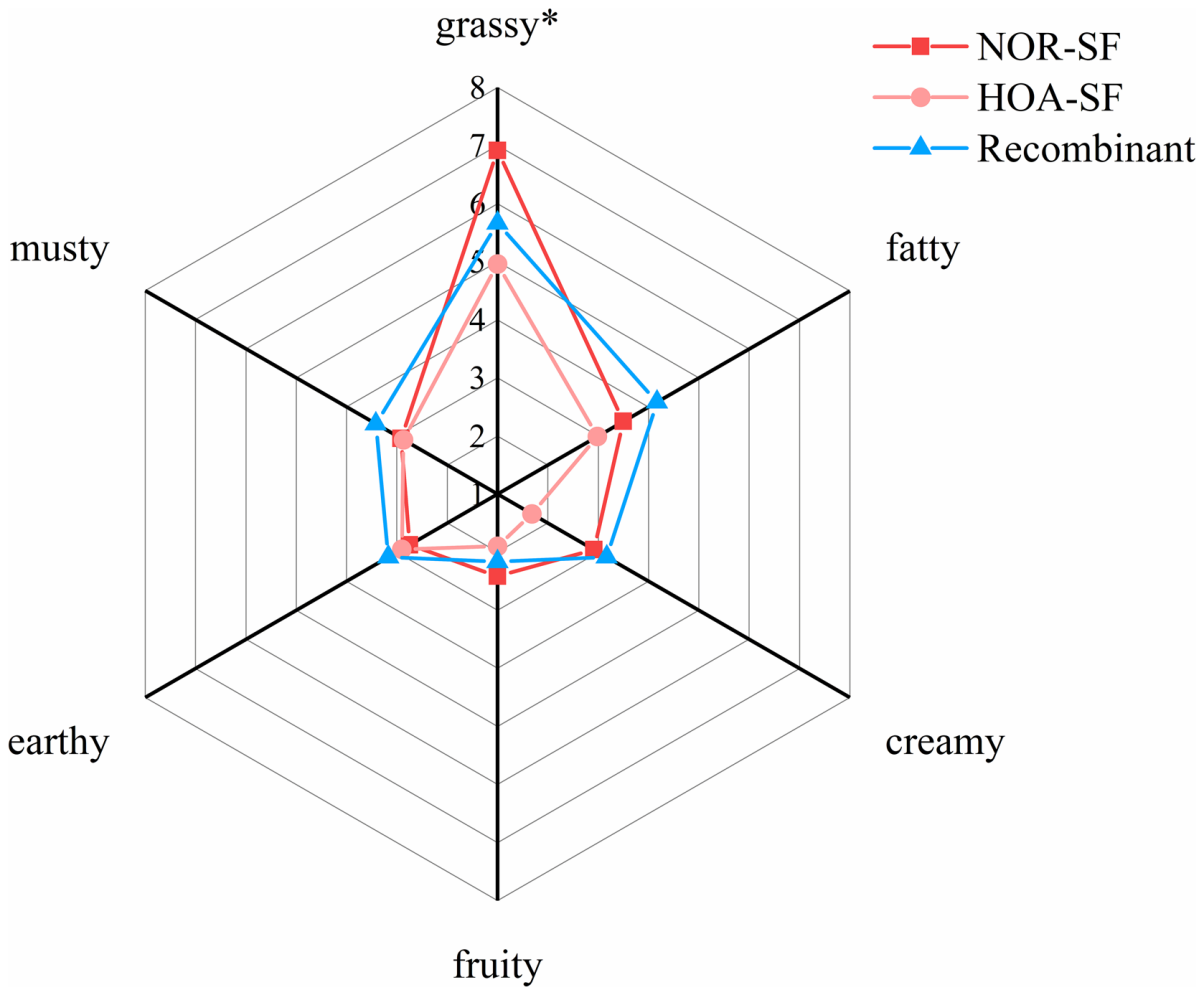
Odor profiles of normal soybean flour, high oleic acid soybean flour, and the recombinant.

QDA was used to characterize the overall odor profile of SF samples. As illustrated in Figure 1, a total of six odor descriptions were evaluated with the attributes of grassy, fatty, creamy, fruity, earthy, and musty. Among these odor notes, HOA-SF showed a relatively lower AI than NOR-SF with the exception of earthy note, indicating the potentiality for off-flavor control of the soybean-based product through editing oleate desaturase genes. It is worthy to point out that significant difference can be only observed in grassy note (*p* < 0.05), which may be the dominated source of beany flavor in agreement with an earlier study (34). This attribute was associated with aldehydes, alcohols, and ketones, derived from lipid oxidation pathway. Therefore, specific volatile compounds responsible for odor differences should be further investigated by molecular sensory science approaches.

### 3.2 Identification of the volatile compounds in NOR-SF and HOA-SF

To anatomize the sensory difference between NOR-SF and HOA-SF, the volatile compounds were identified and quantified. As shown in Table 2, 54 and 39 components were detected in NOR-SF and HOA-SF, respectively. Moreover, total concentration of volatile compounds of NOR-SF was significantly higher than that of HOA-SF (*p* < 0.05). Taking no account of odor activity, these differences could explain why NOR-SF showed a relatively lower AI than NOR-SF. Obviously, alcohols and aldehydes are dominant in both species and concentrations of SF samples. Among which, the hexanal (grassy odor) and 1-octen-3-ol (musty odor) accounted for 74.29 and 51.87% of odor concentration within NOR-SF and HOA-SF, respectively. With regard to their low odor thresholds (1–75 ng/g), hexanal and 1-octen-3-ol, originated from enzymatic/non-enzymatic lipid oxidation, were suggested as the potential contributors to beany flavor of SFs, coinciding with previous research on soybean-based foods (13, 44, 45). These findings provide preliminary evidence to understand the sensory difference between NOR- and HOA-SF from view of component basis. Nevertheless, identification of the odor-active off-flavors is necessary allowing for odor activity.

**Table 2:** Volatile compounds identified in NOR-SF and HOA-SF.

No.	Compounds	CAS	LRI^a^	ID Methods^b^	Concentrations (ng/g)	
					NOR-SF	HOA-SF
Aldehyde						
1	Pentanal	110-62-3	–	MS, O	84.9 ± 3.92	27.98 ± 0.96
2	Hexanal	66-25-1	1,072	MS, LRI, O, S	2877.34 ± 90.34	839.92 ± 27.26
3	Heptanal	111-71-7	1,182	MS, LRI, O, S	85.89 ± 7.11	54.56 ± 6.70
4	(E)-2-Hexenal	6,728-26-3	1,218	MS, LRI, O, S	226.85 ± 8.45	94.63 ± 4.19
5	Octanal	124-13-0	1,289	MS, LRI, O, S	24.53 ± 3.61	47.24 ± 5.10
6	Nonanal	124-19-6	1,396	MS, LRI, O, S	151.69 ± 17.74	363.88 ± 17.13
7	(E)-2-Octenal	2,548-87-0	1,434	MS, LRI, O, S	133.17 ± 4.85	12.17 ± 2.99
8	(E)-2-Nonenal	18,829-56-6	1,546	MS, LRI, O, S	47.52 ± 2.13	–^c^
9	Benzaldehyde	100-52-7	1,533	MS, LRI, O, S	2.24 ± 0.25	–
10	2,4-Nonadienal	6,750-03-4	1713	MS, LRI	12.02 ± 1.39	–
Alcohol						
11	2,2-Dimethyl-1-octanol	2,370-14-1	1,007	MS	5.65 ± 0.55	4.12 ± 0.32
12	2,7-Dimethyl-1-octanol	15,250–22-3	1,094	MS	11.00 ± 1.49	5.16 ± 0.45
13	2,5-Dimethyl-5-hexen-3-ol	67,760-91-2	1,119	MS	1.52 ± 0.06	1.58 ± 0.34
14	cyclobut-1-enylmethanol	89,182-08-1	1,125	MS	70.67 ± 2.71	21.74 ± 3.29
15	1-Penten-3-ol	616-25-1	1,159	MS, LRI, O	63.60 ± 5.19	–
16	2-Methyl-1-butanol	137-32-6	1,209	MS, LRI	6.89 ± 1.02	3.10 ± 0.55
17	3-Methyl-1-butanol	123–51-3	1,210	MS, LRI	9.91 ± 0.99	2.17 ± 0.47
18	1-Pentanol	71-41-0	1,253	MS, LRI, O	63.89 ± 1.34	61.47 ± 9.2
19	1-Hexanol	111-27-3	1,358	MS, O, S	363.09 ± 14.62	116.23 ± 14.18
20	1-Hepten-4-ol	3,521-91-3	1,377	MS	3.88 ± 0.62	1.19 ± 0.31
21	(Z)-3-Hexen-1-ol	928-96-1	1,389	MS, LRI	8.60 ± 0.76	1.26 ± 0.34
22	5-Methyl-3-heptanol	18,720-65-5	1,398	MS	65.31 ± 1.70	–
23	(E)-3-Nonen-1-ol	10,339-61-4	1,405	MS	5.43 ± 0.97	5.86 ± 1.04
24	2,4-dimethyl-3-pentanol	600-36-2	1,437	MS	6.90 ± 0.28	–
25	1-Octen-3-ol	3,391-86-4	1,458	MS, LRI, O, S	4125.62 ± 99.83	372.87 ± 5.32
26	1-Heptanol	111-70-6	1,464	MS, LRI	57.24 ± 1.40	–
27	2-Nitrohept-2-en-1-ol	104,313-51-1	1,549	MS	27.85 ± 0.62	–
28	(E)-2-Octen-1-ol	18,409-17-1	1,625	MS, LRI	35.19 ± 5.82	3.37 ± 0.49
29	3-Decyn-2-ol	69,668-93-5	1,629	MS	7.00 ± 0.73	2.48 ± 0.4
30	1-nonanol	143-08-8	1,670	MS, LRI, O, S	144.34 ± 4.13	44.4 ± 3.29
31	2-Nonen-1-ol	22,104-79-6	1723	MS, LRI	18.64 ± 3.10	1.58 ± 0.28
32	3-Phenyl-1,3-pentanediol	93,306-72-0	1757	MS	87.68 ± 11.30	63.55 ± 8.25
33	Benzyl alcohol	100-51-6	1895	MS, LRI	22.84 ± 3.97	4.49 ± 0.81
34	Phenylethyl alcohol	60-12-8	1936	MS, LRI	18.16 ± 1.97	3.67 ± 0.44
Ketone						
35	2,3-Butanedione	431-03-8		MS, O	1.23 ± 0.31	–
36	1-Penten-3-one	1,629-58-9	1,002	MS, LRI	11.58 ± 1.64	–
37	2,3-Pentanedione	600-14-6	1,042	MS, LRI, O	45.98 ± 6.54	32.82 ± 2.63
38	2-methyl-3-pentanone	565-69-5	1,048	MS	5.71 ± 0.66	1.04 ± 0.23
39	2-Heptanone	110-43-0	1,180	MS, LRI, O, S	0.39 ± 0.06	–
40	(E, E)-3,5-Octadien-2-one	30,086–02-3	1,580	MS, LRI	11.68 ± 1.74	–
41	3,6-Dimethyl-octan-2-one	118,452-32-7	1,678	MS	10.89 ± 1.59	3.41 ± 0.17
42	Maltol	118-71-8	2000	MS, LRI	70.33 ± 8.50	12.96 ± 2.6
Lactone						
43	5-Heptyldihydro-2(3H)-furanone	104-67-6	1719	MS, LRI	6.66 ± 1.71	1.7 ± 0.44
Heterocycle						
44	2-Pentylfuran	3,777-69-3	1,229	MS, LRI, O, S	93.15 ± 2.33	13.59 ± 4.39
Aromatic hydrocarbon						
45	(1-butylpentyl)-benzene	20,216-88-0	1,195	MS	188.16 ± 6.56	81.55 ± 62.32
Acid						
46	Acetic acid	64-19-7	1,455	MS, LRI	30.84 ± 2.33	–
47	(E)-2-Octenoic acid	1871-67-6	1,641	MS	2.68 ± 0.29	1.7 ± 0.21
48	Diisopropyl adipate	6,938-94-9	1881	MS	21.51 ± 3.23	8.44 ± 1.66
49	Nonanoic acid	112-05-0	2,175	MS	24.95 ± 3.48	15.81 ± 1.83
Ester						
50	6-Methylheptyl vinylether	10,573-35-0	1,053	MS	1.37 ± 0.38	-
51	Octadecanoic acid, (2-phenyl-1,3-dioxolan-4-yl) methyl ester	56,599-88-3	1,281	MS	1.25 ± 0.49	0.52 ± 0.11
52	Cyclopropanetetradecanoic acid, 2-octyl-, methyl ester	52,355-42-7	1,427	MS	7.50 ± 1.92	2.12 ± 0.49
53	Stearic acid, 3-(octadecyloxy)propyl ester	17,367-40-7	1,440	MS	3.02 ± 0.55	1.78 ± 0.5
54	Methyl 6-oxoheptanoate	2046-21-1	2,118	MS	10.67 ± 2.28	-
	Total				9426.60 ± 351.55	2338.11 ± 191.68

a Linear retention index (LRI) determined on a DB-Wax column.

b Identification method (ID): MS, identified by MS spectra; LRI, identified by comparison of their LRI with published data; O, identified by comparison of their odor description with the standard compounds via GC–O; S, identified by comparison of their LRI with the standard compounds via GC–MS.

c –, not detected.

### 3.3 Identification of the odor-active off-flavors in NOR-SF

To grasp the sensory difference between NOR-SF and HOA-SF from the perspective of potential odor contributors, the odor-active off-flavors in NOR-SF was identified basing on GC-O-MS analysis. As shown in Table 3, a total of 18 compounds, with AI ranging between 1.00 and 3.33, were detected in olfactory analysis, including 10 aldehydes, five alcohols, two ketones, and one heterocycle. Among them, pentanal (almond, pungent) and 2,3-pentanedione (creamy, caramel) were described with pleasant odor sensation, whereas their AI were only 1.50 and 1.00, respectively, implying the comparatively lower odor activity. Conversely, the other 16 compounds exhibited unpleasant odor sensation regarding the odor profile of SFs, such as grassy, fatty, earthy, etc. Among which, 13 components with AI ≥ 2 were classified as odor-active off-flavor compounds in NOR-SF on the basis of olfactory analysis and previous reports (46, 47). Remarkably, hexanal derived from enzymatic oxidation of linoleic acid was found with the highest AI of 3.33, denoting the dominant contribution to off-flavor sensation (18, 48). Moreover, 2-pentylfuran originated from non-enzymatic lipid oxidation was observed with the second highest AI of 2.80, indicating the relatively higher odor activity. 1-Pentanol was reported for the first time as a volatile off-flavor compound in NOR-SF, originating from the lipoxygenase-catalyzed oxidation of linoleic acid during the grinding process (49). The results furnish viewpoints on the off-flavor sensation of SF in the light of odor activity. With the purpose of focusing on the main contributor to off-flavor, however, differences in odor activity need to be detailed to screen the major off-flavor compounds.

**Table 3:** Identification of the odor-active off-flavors in NOR-SF by AI.

No.	Compounds	CAS	LRI	Identification Methods	Odor description^a^	AI^b^
1	Pentanal	110-62-3	–	MS, O	Almond, pungent	1.50
2	2,3-Pentanedione	600-14-6	1,042	MS, RI, O	Creamy, caramel	1.00
3	Hexanal	66-25-1	1,072	MS, RI, O, S	Grassy, leaf	3.33
4	1-Penten-3-ol	616-25-1	1,159	MS, RI, O	Beany, leaf	1.00
5	2-Heptanone	110-43-0	1,180	MS, RI, O, S	Soapy, fatty	2.14
6	Heptanal	111-71-7	1,182	MS, RI, O, S	Fatty, grassy	2.33
7	(E)-2-Hexenal	6,728-26-3	1,218	MS, RI, O, S	Grassy, leaf	1.75
8	2-Pentylfuran	3,777-69-3	1,229	MS, RI, O, S	Green, beany, butter	2.80
9	1-Pentanol	71-41-0	1,253	MS, RI, O, S	Fermented, Grassy	2.00
10	Octanal	124-13-0	1,289	MS, RI, O, S	Fatty, grassy	2.14
11	1-Hexanol	111-27-3	1,358	MS, O, S	Beany, vegetable	2.33
12	Nonanal	124-19-6	1,396	MS, RI, O, S	Fatty, soapy	2.00
13	(E)-2-Octenal	2,548-87-0	1,434	MS, RI, O, S	Grassy, fatty	2.00
14	1-Octen-3-ol	3,391-86-4	1,458	MS, RI, O, S	Mushroom, musty	2.25
15	(E)-2-Nonenal	18,829-56-6	1,546	MS, RI, O, S	Grassy, fatty	2.33
16	Benzaldehyde	100-52-7	1,533	MS, RI, O, S	Almond, caramel	2.00
17	1-Nonanol	143-08-8	1,670	MS, RI, O, S	Earthy, grassy	2.00
18	2,4-Nonadienal	6,750-03-4	1713	MS, RI, O	Fatty, grassy	1.00

a Odor description described by sensory assessors in GC-O-MS analysis.

b Odor intensity (AI): 1 = low intensity; 2 = moderate intensity; 3 = high intensity; and 4 = very high intensity.

### 3.4 Identification of the major odor-active off-flavors in NOR-SF

**Figure 2:**
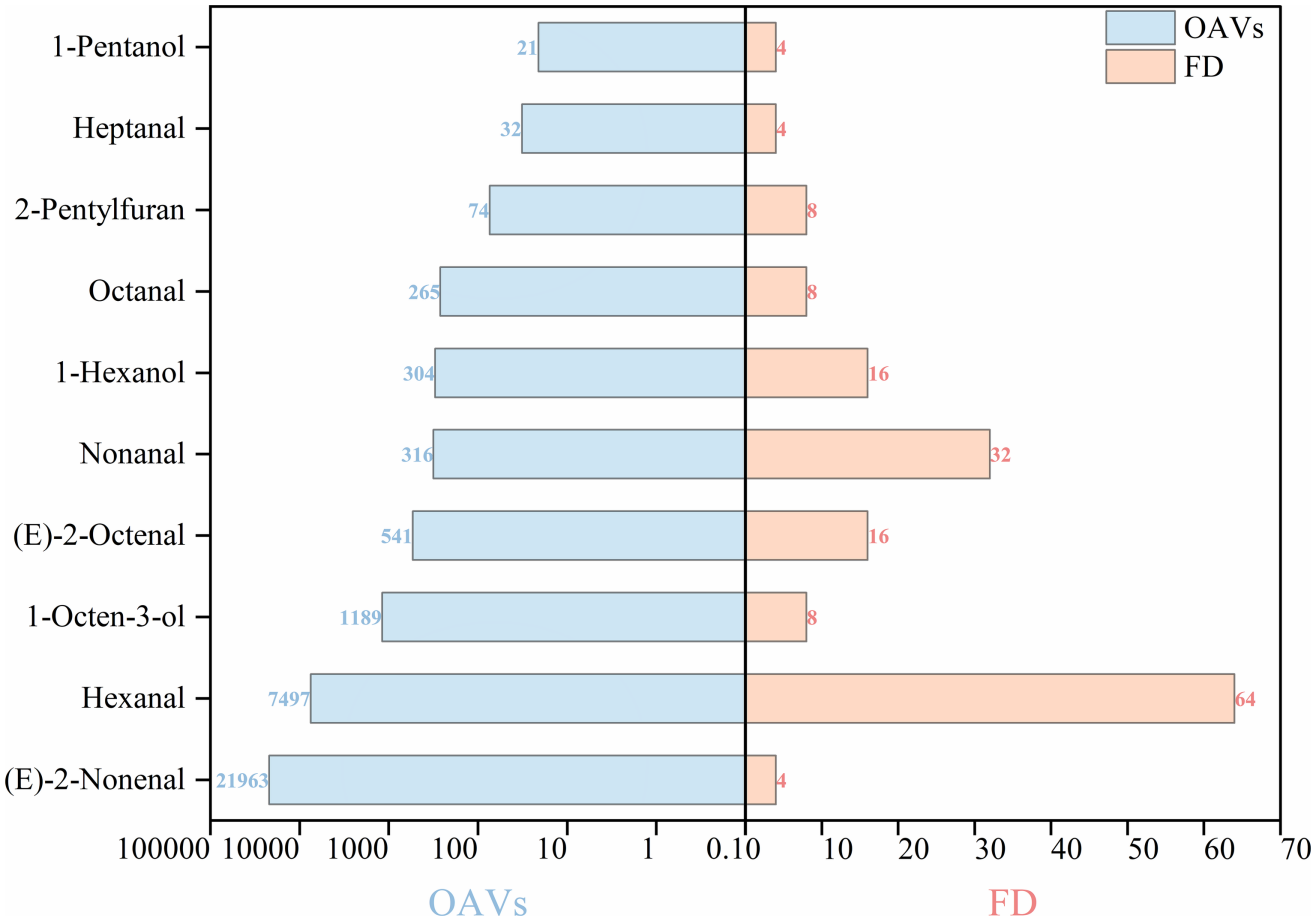
Odor activity values and flavor dilution values of key odor-active off-flavor compounds in normal soybean flour.

To provide insights into the main odor contributors to the off-flavor sensation of NOR-SF, odor activity of the odor-active off-flavor compounds was ulteriorly assessed via a revised ADA (Table 1). As illustrated in Figure 2, 10 volatile compounds, with FD ranging from 4 to 64, were identified as the major odor-active off-flavors in NOR-SF. Similar to the results of AI, hexanal showed the highest FD of 64, suggesting that grassy note might be the most important odor attribute of off-flavor profile. Octanal derived from enzymatic oxidation of linolenic acid was found with the second highest FD of 32, indicating that fatty note also plays an important role to off-flavor sensation.

Furthermore, OAVs of the above 13 odor-active off-flavor compounds were calculated to validate the odor activity evaluations. Before that, the 13 compounds were requantified through SIDA in consideration of matrix effect on odor release. Both isotopically labeled and unlabeled analytes, sharing the same physicochemical properties, were employed to improve the recovery rate, thereby eliminating matrix effects associated with sample preparation (30, 50). As shown in Table 1, the calibration curves of the 13 isotope-labeled analytes showed good linearity within the selected concentration range, with correlation coefficients (*R*^2^) higher than 0.99. In addition, the LOD and LOQ for each analyte were much lower than its measured concentration, indicating that the developed method had good detection sensitivity. Overall, the calibration curves established for SIDA showed accurate and reliable performance for all 13 compounds. Correspondingly, the 10 major odor-active off-flavors were found with OAVs > 1, whereas the other three off-flavors were observed with OAVs < 1. It was suggested that olfactory analysis combining AI and FD was an effective strategy to identify the odor-active off-flavor, and SIDA suited for quantification in complex food matrices comparing to conventional standard method. However, the odor activity evaluations based on OAVs do not entirely chime with those based on FD. The grassy (E)-2-nonenal was found with the highest OAV of 21,963, followed by hexanal (7497) and 1-octen-3-ol (1189). The above differences were due to several reasons, including (a) the OAVs were calculated in terms of the odor threshold values in water, rather than those in soy protein matrix; (b) the complex interactions among volatile compounds and food matrices were hard to consider, comprising volatile compound–volatile compound interactions, nonvolatile component–nonvolatile component interactions, and volatile compound–nonvolatile component interactions (25).

### 3.5 Identification of the key odor-active off-flavors in NOR-SF

To verify the odor contributions of the 10 major odor-active off-flavors and screen the key odor-active off-flavors, odor recombination and omission tests were conducted progressively. As shown in Figure 1, six odor descriptions of an odor recombination model were rated in agreement with NOR-SF. Among them, musty, earthy, creamy, and fatty notes of the recombination model were rated slightly higher than those of NOR-SF, however, the differences were not significant (*p* > 0.05). Similarly, no significant difference (*p* > 0.05) was observed in the fruity note, whereas its intensity in the recombination model was rated slightly lower than that in NOR-SF. These findings revealed that the musty, earthy, creamy, fatty, and fruity notes of NOR-SF can be simulated through the 10 major odor-active off-flavors. Noteworthily, grassy note of odor reconstitutes showed a relatively lower intensity than that of NOR-SF. The above differences were due to several reasons, including (a) more odor information need be profiled through comprehensive two-dimensional gas chromatography (GC × GC); (b) volatile compounds with relatively lower odor activity contribute to odor perception, working together with the 10 major odor-active off-flavors (suppression, synergism, and antagonism); (c) multi-interactions among volatile compounds and food matrices struggle to be incorporated into current molecular sensory science approaches (25, 51, 52). In general, the recombination model simulates the odor profile of NOR-SF reasonably well, especially in musty, earthy, creamy, fatty, and fruity notes, thereby it could be applied to prepare the odor omission models.

To confirm the odor contribution of individual odor-active off-flavor compound, a triangle test was performed, for which 10 omission model solutions were prepared to compare with the complete recombination model solution, respectively. As shown in Table 4, significant differences could be observed in all of the 10 triangle tests (*p* < 0.05). The above 10 major odor-active off-flavors were therefore identified as key off-flavor compounds resulted from their critical odor contribution to NOR-SF. However, the significance level differed between the 10 omission models and recombination model. With respect to extremely significant difference (*p* < 0.001), the sensory panel could discriminate the omission model solution, when hexanal, heptanal, and octanal were omitted, respectively. These suggest key off-flavor from lipid oxidation may play an important role in grassy and fatty notes of NOR-SF. It is noteworthy that the *n* value of omission test 1 is 12, which means that all of the 12 assessors can accurately recognize the recombination model solution with omission of hexanal. This proves the critical odor contribution of hexanal to soybean-based food once again, in accordance with previous flavor research on SPI, soy milk, and soy yogurt (18, 49, 53, 54). Moreover, highly significant differences were reported (*p* < 0.01), when 1-pentanol, nonanal, and 1-octen-3-ol were omitted, respectively. These findings indicate the importance of fatty and musty notes to off-flavor profile of SF, in agreement with the odor profile of SPIs (34).

**Table 4:** Identification of the key odor-active off-flavors in NOR-SF by omission tests.

No.	Compound omitted	*N* ^a^	Significance^b^
1	Hexanal	12	***
2	Heptanal	10	***
3	2-Pentylfuran	8	*
4	1-Pentanol	9	**
5	Octanal	11	***
6	1-Hexanol	8	*
7	Nonanal	9	**
8	(E)-2-Octenal	8	*
9	1-Octen-3-ol	9	**
10	(E)-2-Nonenal	8	*

a n, the number of people among the 12 participants in the triangle test who gave correct evaluations.

b The significance is evaluated based on the results of the three-point test inspection table: NS, no significant difference; _, not contained in this model; ***, 0.1% significance level; **, 1% significance level; *, 5% significance level.

### 3.6 Dynamic changes in key odor-active off-flavors throughout the process of soybean-based products

**Figure 3:**
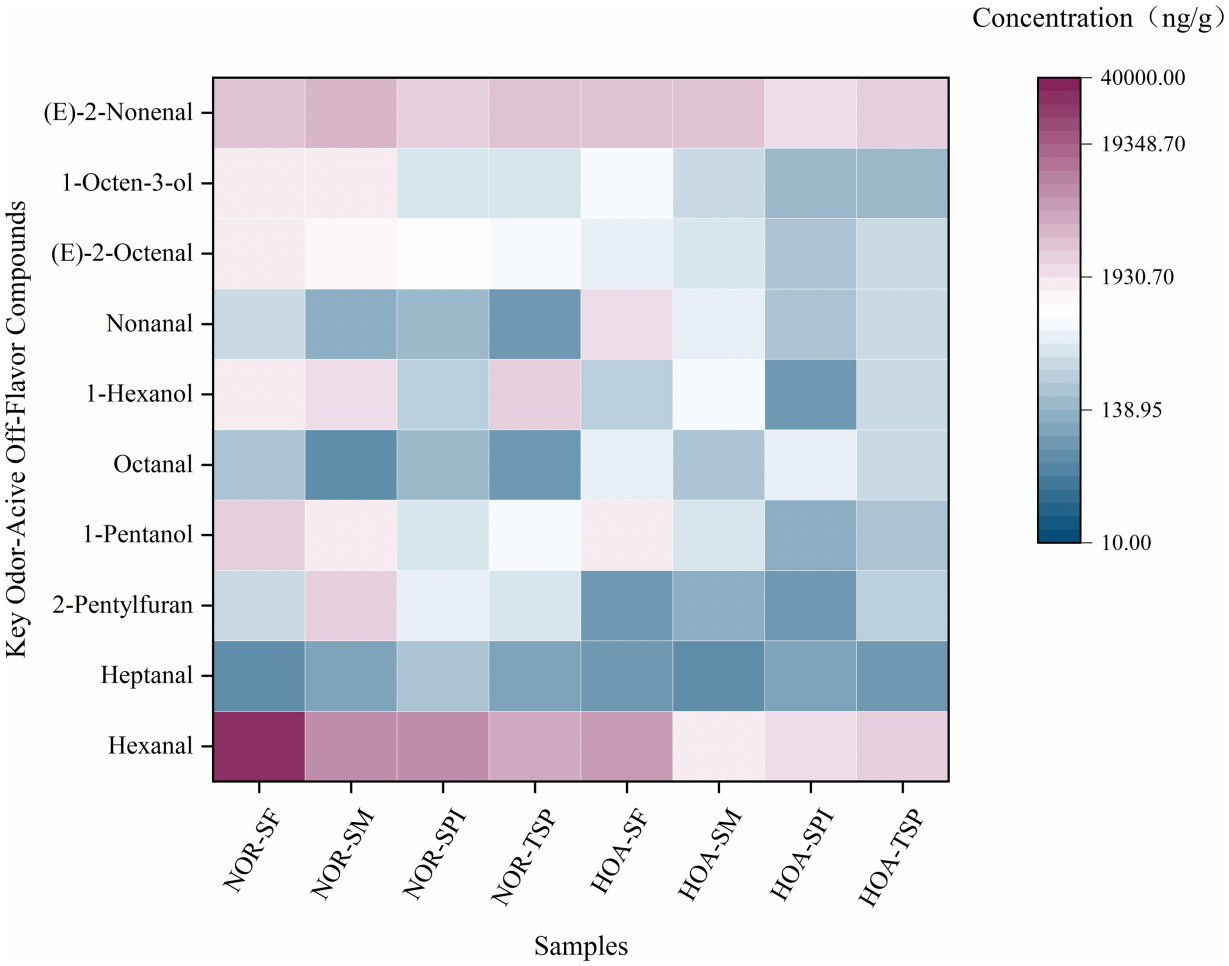
Heatmap of key off-flavor compound concentrations in eight protein samples.

**Figure 4:**
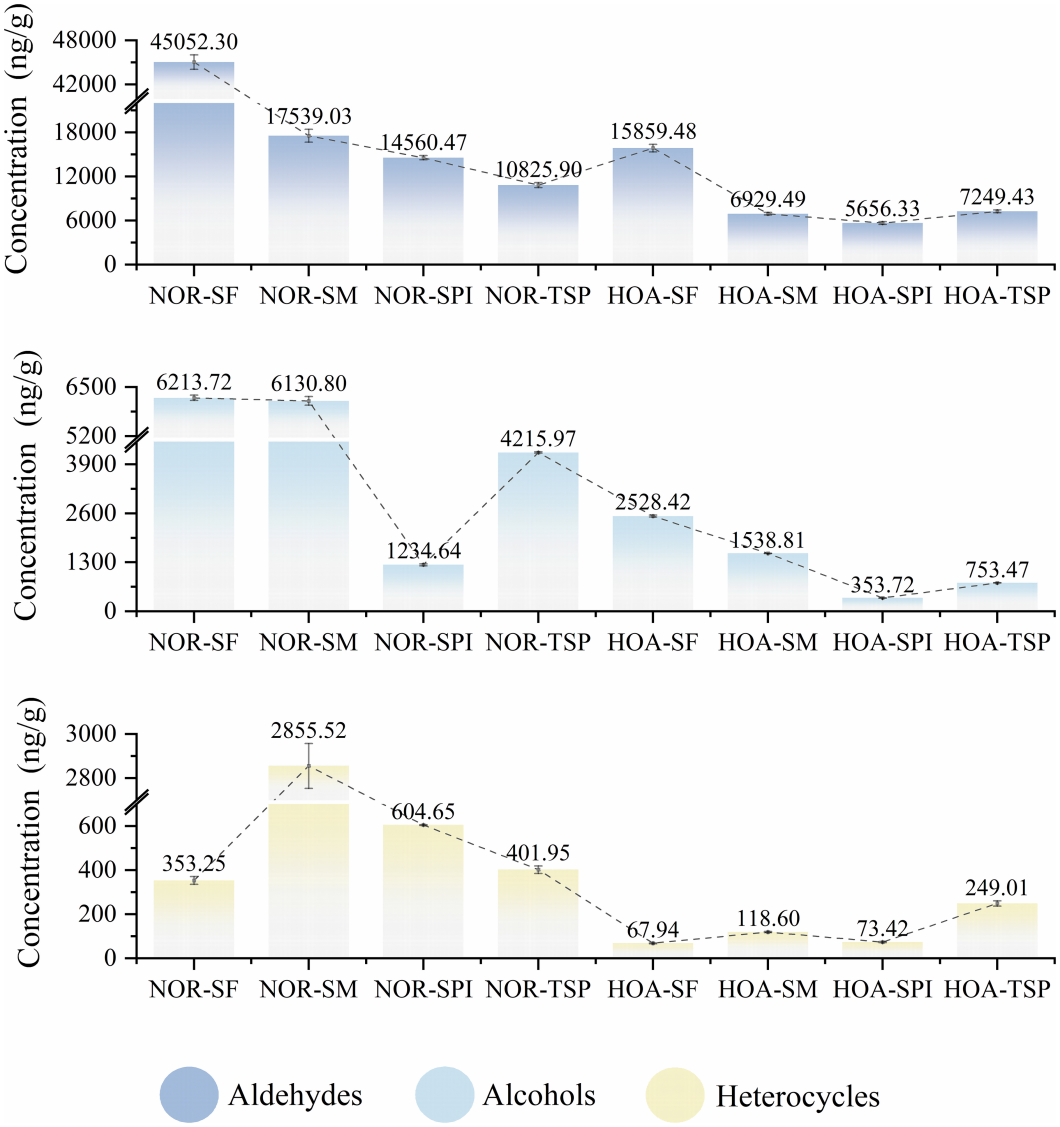
Concentrations of aldehydes, alcohols and heterocycles in eight soybean protein samples.

To expand application of the key odor-active off-flavors in quality control of soybean-based food, SIDA was used to quantify the above 10 essential components in SF, SM, SPI, and TSP, which were manufactured from NOR and HOA. As illustrated in Figure 3, the heatmap clearly indicates significant differences in the concentrations of the 10 key off-flavor compounds. Among them, the compound with the highest concentration was hexanal, reaching approximately 39,000 ng/g in NOR-SF, while the lowest concentrations were observed for heptanal in HOA-SM and octanal in NOR-SM, at 54.59 and 53.71 ng/g, respectively, with a difference of over 700 times. Owing to their low threshold values, aldehydes are considered the most important class of compounds among the key off-flavors (55). Second, by comparing the concentrations of key off-flavor compounds in NOR and HOA samples during processing, we found that the concentrations of most key off-flavor compounds in HOA soybean protein samples were lower than those in NOR soybean protein samples. For example, hexanal, 1-octen-3-ol, and (E)-2-octenal were present at lower levels. The lower formation of several lipid-derived odorants in HOA-derived products may be related to the known fatty acid characteristics of high-oleic soybean, which generally contains higher oleic acid and lower linoleic and linolenic acids than NOR soybeans (56, 57). As shown in Figure 4, the total concentrations of aldehydes, alcohols, and heterocycles in the key odor-active off-flavor compounds were higher in the NOR samples than in the HOA samples. This observation is consistent with the findings of Bancroft et al., who reported similar results in NOR and HOA soybean milk (13). However, the concentrations of nonanal, octanal, and heptanal were higher in the HOA soybean samples than in the NOR samples. This may be attributed to the increased oleic acid content, which enhances the thermal-oxidative degradation of oleic acid via the alkoxy radical pathway, leading to excessive formation of compounds such as octanal and nonanal (58).

Throughout the life cycle of soybean-based food products, changes in the concentration of key off-flavor compounds can be divided into three main stages. In the first stage, when soybeans are pressed into SM, most of the oil is removed, resulting in a decrease in the fatty acid content, and consequently, a reduction in the levels of off-flavor compounds. As shown in Figure 3, most of the 10 key odor-active compounds exhibited a decreasing trend. However, the concentrations of (E)-2-nonenal, 1-octen-3-ol, and heptanal increased significantly in NOR-SM, whereas the concentrations of 1-hexanol and 2-pentylfuran increased markedly in both NOR- and HOA-SM. Second, during the alkali solubilization and acid precipitation stages for isolating soybean protein from SM, the concentrations of most key odor-active compounds gradually decreased, as shown in Figure 4. Finally, during the extrusion process to form NOR-TSP, the concentrations of several key compounds, such as (E)-2-nonenal, 1-pentanol, and 1-hexanol, increased significantly, whereas most of the others continued to decline. Similarly, during the extrusion of HOA soybean protein, the concentrations of most key odor-active compounds decreased markedly. However, octanal and heptanal levels increased significantly by approximately 2–3 times in both NOR- and HOA-SPIs and TSPs. This increase is likely because of the high-temperature extrusion conditions, which intensify oleic acid oxidation through alkoxy radical pathways, resulting in the generation of more aldehyde compounds.

The integration of volatile profile, FD, OAVs, and SIDA-based quantitative data further revealed the dynamic changes in off-flavor compounds during progressive soybean processing. The key odorants showed a clear pattern related to processing. During oil removal and protein isolation, the contents of several lipid-derived compounds decreased, but increased markedly after extrusion. This pattern suggests that early processing steps may remove part of the volatile fraction or reduce the availability of odor-active compounds (59). Extrusion may then promote secondary formation and release of these compounds, as thermal and mechanical effects can accelerate lipid oxidation and alter the protein matrix (60). The evolution of these odorants also indicates that soybean off-flavor is generated by a group of chemically and sensorially related compounds. Aldehydes, alcohols, and furans showed closely linked changes because they are mainly derived from the oxidation and degradation of unsaturated fatty acids (61). Hexanal, 2-pentylfuran, 1-octen-3-ol, (E)-2-octenal, and (E)-2-nonenal represented the main oxidation-related odorants and contributed jointly to grassy, fatty, green, musty, and earthy odor (18). Among them, hexanal showed a high concentration and strong sensory relevance. 2-Pentylfuran and 1-octen-3-ol reflected typical earthy and musty odor characteristics. In comparison, unsaturated aldehydes such as (E)-2-octenal and (E)-2-nonenal were present at relatively lower concentrations, but they showed strong odor activity because of their low odor thresholds. Therefore, these compounds may serve as representative odorants that reflect the evolution of off-flavor during soybean processing.

Overall, these results suggest that off-flavor control in soybean-derived products should focus on the coordinated regulation of lipid oxidation-derived odorants. The combined changes in aldehydes, alcohols, and furans provide a more systematic explanation for the formation and evolution of undesirable flavor during processing. This integrated interpretation also provides a basis for process-oriented flavor control, including raw material selection, oxidation inhibition, extrusion optimization, and regulation of protein–odorant interactions. For flavor control, soybean materials with more favorable lipid compositions, such as lower levels of linoleic and linolenic acids, may help reduce the precursor content of lipid-derived off-flavor compounds. In addition, the sharp increase in several key odorants after extrusion indicates that thermal–mechanical processing is a critical control point. Moderate extrusion temperature, shorter residence time, and reduced oxygen exposure may help limit secondary oxidation and off-flavor release (62, 63). Natural antioxidants or enzyme-inactivation treatments may also be used to suppress lipid oxidation at earlier processing stages. Since soy protein structure changes during isolation and extrusion, these changes may affect the binding and release of volatile compounds. Future studies should further explore protein–odorant interactions as a complementary strategy for improving flavor quality.

### 3.7 Orthogonal partial least-squares discriminant analysis of the soybean-based products

**Figure 5:**
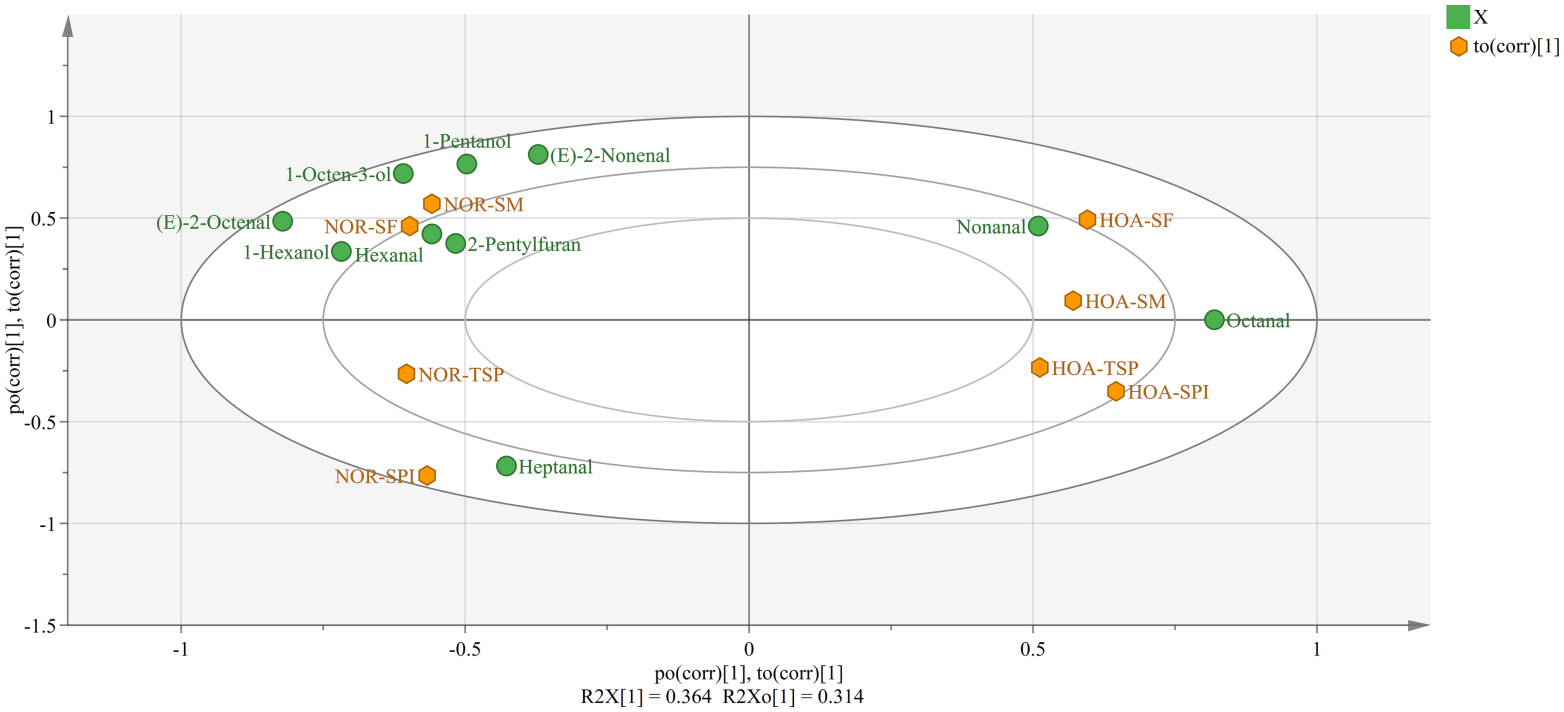
Biplot of orthogonal partial least-squares discriminant analysis for eight soy protein varieties and their concentrations.

Using the concentrations of 10 key off-flavor compounds as the dependent variables and four processing methods, along with two soybean protein samples as the independent variables, OPLS-DA analysis (Figure 5) was used to distinguish between the two varieties and processed samples. The fitting index for the independent variables (*R*^2^*x*) was 0.947, that for the dependent variables (*R*^2^*y*) was 0.996, and that for the model prediction index (*Q*^2^) was 0.978. The *R*^2^ and *Q*^2^ values exceeded 0.5, indicating that the model’s fit was acceptable (64). As shown in Figure 5, the contributions of principal components 1 and 2 were 36.4 and 31.4%, respectively, for a total contribution of 67.8%. Notably, the concentration variations of heptanal, nonanal, and octanal differed from those of other key off-flavor compounds, corroborating the findings observed in the aforementioned heatmap. Additionally, the heptanal concentration had a more significant impact on NOR-SPI and TSP, whereas nonanal exhibited a stronger correlation with HOA-SF and SM. However, octanal affected all HOA soybean protein samples concurrently.

**Figure 6:**
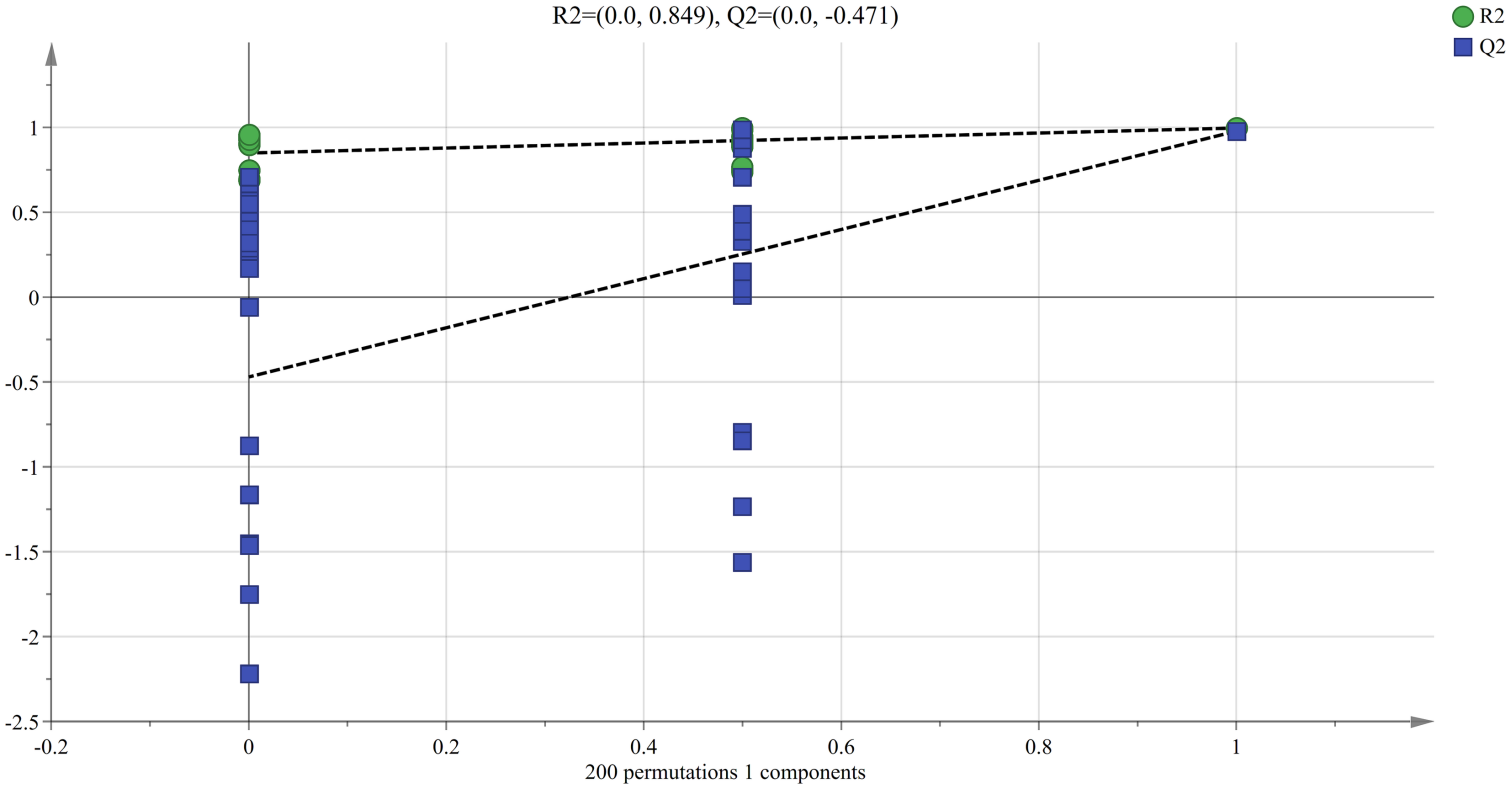
Permutation test of orthogonal partial least-squares discriminant analysis model.

Following 200 permutation tests, as shown in Figure 6, the intercept of the *Q*^2^ regression line with the *y*-axis was below zero, indicating that the model was free from overfitting, validating its robustness. These results suggest that the model can reliably differentiate between soybean protein sample types and processing methods. Moreover, this finding underscores the pivotal role of key off-flavor compounds throughout the life cycle of soybean-based food processing.

## 4 Conclusion

This study presents the potential contributors to off-flavor profile of SF through a collaborative strategy based on molecular sensory science approaches, thereby revealing the dynamic alterations in key off-flavor compounds throughout the step-by-step process. Basing on the AIs in olfactory analysis, 13 components with AI ≥ 2 were suggested as the odor-active off-flavor compounds in NOR-SF. Among them, 10 compounds with FD ≥ 4 and OAVs >1 were considered as the major odor-active off-flavors, namely, hexanal, heptanal, 2-pentylfuran, 1-pentanol, octanal, 1-hexanol, nonanal, (E)-2-octenal, 1-octen-3-ol, and (E)-2-nonenal. Odor recombination and odor omission models were prepared to verify the odor contributions of the 10 major odor-active off-flavors. Significant differences were observed in all of the 10 triangle tests (*p* < 0.05), revealing that the 10 off-flavors were the key off-flavor in SF. Furthermore, dynamic changes in key odor-active off-flavors throughout the process of soybean-based products, manufactured from NOR and HOA were profiled. The results showed that off-flavor formation in soybean-based products is mainly associated with the coordinated changes of lipid oxidation-derived aldehydes, alcohols, and furans. The lower levels of several key odorants in HOA products further suggest the potential of controlling off-flavor through the regulation of fatty acid composition, such as editing oleate desaturase genes. These findings provide a more comprehensive understanding of the off-flavor profile and its evolution in soybean-based products, and offer useful guidance for raw material selection, process optimization, and flavor quality improvement.

## Data Availability

The original contributions presented in the study are included in the article/supplementary material, further inquiries can be directed to the corresponding authors.
